# Effects of dietary protein levels on performance, nitrogen excretion, and odor emission of growing pullets and laying hens

**DOI:** 10.1016/j.psj.2023.102798

**Published:** 2023-05-20

**Authors:** Yun-Ji Heo, Jina Park, Yoo-Bhin Kim, Byung-Yeon Kwon, Da-Hye Kim, Ju-Yong Song, Kyung-Woo Lee

**Affiliations:** Department of Animal Science and Technology, Sanghuh College of Life Sciences, Konkuk University, Gwangjin-gu, Seoul 05029, South Korea

**Keywords:** laying hen, pullet, crude protein level, egg production, nitrogen excretion

## Abstract

The objective of this study was to determine the effects of dietary crude protein (**CP**) levels on production performance, nitrogen balance, and odor emission of excreta in growing pullets and laying hens from 13 to 32 wk of age. Two hundred and forty pullets (Hy-Line Brown) were randomly assigned to 1 of 4 dietary groups with 10 replicates per group, and 6 birds per replicate. Experimental diets were formulated to contain 4 graded CP levels in the diets of pullets ranging from 180, 160, 140, and 120 g/kg of diet during 13 to 18 wk (phase 1) and in the diets of laying hens from 190, 170, 150, and 130 g/kg of diet during 19 to 32 wk (phase 2). The limiting amino acids including lysine, methionine, and threonine were supplemented to maintain constant equal amino acid concentrations in all experiment diets. In phase 1, decreasing dietary CP levels did not affect growth performance but increased (linear and quadratic effect, *P* < 0.05) the relative abdominal fat contents and triglyceride concentration in serum samples. High-density lipoprotein cholesterol in serum samples decreased as the CP levels decreased in the diets of pullets. Dietary CP levels quadratically increased (*P* < 0.05) the villus height and the villus height to crypt depth ratio but did not affect tibia traits and relative organ weights in pullets at 18 wk. Apparent digestibility of dry matter and ether extract increased with decreasing dietary CP levels in pullets. Graded CP levels linearly increased the digestibility of dry matter, CP, and ether extracts but lowered that of crude ash in laying hens. Nitrogen excretion was linearly decreased (*P* < 0.05) as the dietary CP levels decreased in both pullets and laying hens. Dietary CP levels only affected carbon dioxide emission in pullets. In phase 2, dietary CP levels did not affect growth performance and the ages at first egg laying and to reach 50% egg production in laying hens. However, egg weights were decreased (linear and quadratic effect, *P* < 0.05) as the dietary CP level decreased in laying hens. Increasing dietary CP levels increased Haugh unit at 26 wk but lowered corticosterone concentrations in yolk samples at 22 wk. Collectively, this study shows that dietary CP levels could be decreased to reduce nitrogen excretion without adverse effects on performance and egg quality of growing pullets and laying hens.

## INTRODUCTION

In order to maximize growth and laying performance there must be a sufficient supply of dietary protein (Baker, 2009). Protein is needed for growth, development, health, and maintenance (Hou et al., 2015). On the other hand, excess crude protein (**CP**) in diets can lead to increased nitrogen (**N**) excretion and odor emission as undigested and unabsorbed CP in addition to uric acids as the end-product of protein metabolism was simultaneously excreted in fecal droppings (Chalova et al., 2016). Gas pollutants responsible for global warming in livestock industry are odors from excreta (Blanes-Vidal et al., 2012). To solve this problem, many technologies are being developed, which can be divided into 2 categories, that is, technological strategy and nutritional intervention (Patterson and Adrizal, 2005). It is well acknowledged that protein content in the feed is closely correlated with N emissions in excreta (Nahm, 2003; Latshaw and Zhao, 2011). Poultry excretes N in the form of elements such as urea, AA, and creatine but generally uric acid. All are converted to produce odors like ammonia by litter microbes (Ferguson et al., 1998). All these scenarios led to increased concentrations of environment gases including ammonia or amines which are responsible for eutrophication, ecosystem disturbance, and air pollution (Wang et al., 2017). As a result, the interests in reducing the CP content of feeds have re-emerged. Therefore, low protein diets are widely acknowledged nutritional intervention to reduce ammonia emissions and also reduce feed costs by reducing expensive protein source ingredients in the complete diet (Laudadio et al., 2012). It should be, however, kept in mind that the balance between adequacy in nutritional levels and production performance must be considered when CP levels in poultry feed are lowered.

An earlier study showed that low protein levels did not affect growth performance and egg production (Ji et al., 2014). It is thus expected that lowering CP levels in the diets of chickens would be an effective nutritional approach to minimize the nutrient loadings into the environment. Although low CP diets have been known to affect the performance and egg quality of pullets and laying hens, no studies have been tested whether low CP diets could affect organ development, tibia characteristics, and ileal morphology of pullets, and N excretion and odor emission of both pullets and laying hens. In this study, dietary CP levels gradually lowered by a 2%-point decrease from 18 to 12% for pullets and 19 to 13% for laying hens. Thus, the objective of this study was to investigate the effects of dietary CP levels on performance, organ development, N balance, and odor emission of pullet and laying hens.

## MATERIALS AND METHODS

### Animal Care

All protocols were reviewed and approved by the Institutional Animal Care and Use Committee in Konkuk University (KU 21081).

### Animals Experimental Design and Diets

An experiment had 2 feeding phases (i.e., growing and laying phase) from 13 to 18 wk (phase 1) and from 19 to 32 wk (phase 2), respectively. Two hundred and forty 13-wk-old Hy-Line Brown pullets were kept in 3-tiered cages and randomly assigned to 1 of 4 dietary treatments with 10 replicates per treatment. Each cage (45 × 45 × 45 cm) was equipped with a plastic trough feeder and a nipple waterer. Each cage housed 2 pullets and 3 adjacent cages (6 pullets per replicate) considered a replicate. Feed and water were provided ad libitum. The lighting program employed 12L:12D during 13 to 17 wk and 13L:11D at 18 wk. The light exposure was gradually increased from 13L:11D at 18 wk to reach 16L:8D at 24 wk.

Four experimental diets were prepared to contain different CP levels from 18 to 12% in phase 1 (Table 1) and from 19 to 13% in phase 2 (Table 1). The experimental groups that were fed on diets containing 18, 16, 14, and 12% CP during the growing phase were given the feeds containing 19, 17, 15, and 13% CP during the laying phase. To formulate the experimental diets in each phase, 2 CP diets with 18 or 12% in phase 1 and 19 and 13% in phase 2 were prepared and they were proportionally mixed with the following ratios: 67:33 and 33:67 to produce 16 and 14% CP levels in phase 1 and 17 and 15% CP levels in phase 2. Metabolizable energy and the selected essential amino acids in the diets were formulated to meet or exceed the requirements recommended by the Hy-Line Brown Management Guide (Hy-Line, 2018). All diets were prepared in a mash form. All birds were individually weighed for their body weights at 13, 18, and 32 wk to measure the body weight gain at phases 1 and 2. The amount of feed offered was recorded daily and the amount of feed left in the feeders was recorded fortnightly.

**Table 1:** Ingredients and calculated chemical composition of experimental diets with several crude protein (CP) levels in phase 1 (13–18 wk) and 2 (19–32 wk), as-fed basis.

Item	Phase:	One (13–18 wk)				Two (19–32 wk)			
	Protein levels:	18	16^1^	14^1^	12	19	17^2^	15^2^	13
Ingredient composition, %									
Corn		68.05	70.68	73.25	75.84	50.24	55.39	60.53	65.66
Soybean meal, 46% CP		27.23	20.41	13.60	6.80	25.34	21.92	18.51	15.09
Tallow		1.02	0.68	0.34	-	5.50	5.09	4.68	4.27
Wheat middling		-	4.05	8.09	12.14	-	-	-	-
Corn gluten meal		-	-	-	-	5.09	3.39	1.69	-
Limestone		1.55	1.61	1.67	1.73	11.18	11.20	11.23	11.25
MCP		1.40	1.39	1.39	1.38	1.60	1.62	1.63	1.65
Sodium chloride		0.20	0.20	0.21	0.21	0.30	0.30	0.31	0.32
Choline chloride		-	-	-	-	0.10	0.10	0.10	0.10
Vitamin premix^3^		0.20	0.20	0.20	0.20	0.20	0.20	0.20	0.20
Mineral premix^3^		0.25	0.25	0.25	0.25	0.25	0.25	0.25	0.25
L-lysine·HCl, 78.4%		-	0.20	0.41	0.62	0.10	0.21	0.31	0.42
DL-methionine, 99%		0.10	0.13	0.17	0.20	0.10	0.14	0.18	0.22
L-threonine, 99%		-	0.09	0.19	0.28	-	0.08	0.16	0.24
Chemical composition									
Dry matter		87.43	87.40	87.37	87.34	89.56	89.41	89.26	89.11
AMEn, kcal/kg		2,850	2,850	2,850	2,850	2,800	2,800	2,800	2,800
Crude protein		18.12	15.95	14.00	12.13	19.28	17.00	15.37	13.08
Energy to protein ratio		158	178	204	238	147	165	187	215
Ether extract		3.87	3.67	3.48	3.29	7.99	7.58	7.17	6.77
Ash		5.96	5.78	5.61	5.44	15.22	15.07	14.93	14.79
Calcium		0.90	0.90	0.90	0.90	4.29	4.29	4.29	4.29
Total phosphorus		0.73	0.72	0.71	0.70	0.79	0.78	0.77	0.76
Available phosphorus		0.45	0.45	0.45	0.45	0.50	0.50	0.50	0.50
Lysine		0.94	0.94	0.94	0.94	1.00	1.00	1.00	1.00
Methionine		0.39	0.39	0.39	0.39	0.42	0.42	0.42	0.42
Threonine		0.68	0.68	0.68	0.68	0.70	0.70	0.70	0.70

Abbreviation: MCP, monocalcium phosphate.

1 Two diets that contain CP concentrations at 16 and 14% were prepared by the proportional mixing of 18 and 12% of CP diets at the following ratios: 66.7 to 33.3 and 33.3 to 66.7, respectively.

2 Two diets that contain CP concentrations at 17 and 15% were prepared by the proportional mixing of 19 and 13% of CP diets at the following ratios: 66.7 to 33.3 and 33.3 to 66.7, respectively.

3 Vitamin and mineral premixes provided the following quantities per kg of complete diets: vitamin A, 36,000 IU; vitamin D_3_, 6,000 mg; vitamin E, 160 IU; vitamin K, 10 mg; vitamin B_1_, 10 mg; vitamin B_2_ 40 mg; vitamin B_6_, 16 mg; vitamin B_12_, 0.12 mg; biotin, 0.40 mg; pantothenic acid, 100 mg; niacin, 180 mg; Fe, 200 mg; Mn, 100 mg; Zn, 31.25 mg; Cu, 75 mg; I, 0.63 mg; and Se, 0.50 mg.

### Sample Collection

On the last day of 18 wk, 7 birds per treatment were randomly euthanized by an overdose of carbon dioxide and sampled for blood. Serum samples were separated by centrifugation (200 × *g* for 15 min) and stored at −20°C until serum analyses. Immediately after blood sampling, abdominal fat, liver, spleen, bursa of Fabricius, ovary, oviduct, large yellow follicle, small intestine, and tibia samples were collected. A 1-cm long proximal ileal segment was collected and immediately fixed with 10% neutral buffered formalin for histological analysis. Tibia samples from the right leg were obtained by manually removing the attached meat and cartilage.

### Measurements of Serum Biochemical Profiles, Ileal Morphology, Organ Weights, and Tibia Characteristics

In phase 1, all serum samples were analyzed for total cholesterol, triglyceride, glutamic oxaloacetic transaminase, glutamic pyruvic transaminase, albumin, high-density lipoprotein cholesterol, total protein, and uric acid using an automatic blood chemical analyzer (Film DRI CHEM 7000i, Fuji film Co., Tokyo, Japan). Organ weights were recorded and expressed as relative organ weights. Large yellow follicles over 0.5 cm were counted and recorded. The fixed ileal samples were sectioned at 4 μm thickness, mounted on slides, and stained with standard hematoxylin-eosin solution. Villus height and crypt depth were observed in 7 well-oriented intact villi at 40× magnification using an Olympus BX 43 digital microscope (Olympus, Tokyo, Japan) and photographed using a digital camera (eXcope T500, Olympus, Tokyo, Japan). The width and length of the tibia were measured using a vernier caliper. Tibia breaking strength was measured using an Instron (Instron Universal Testing Machine Mo. 3342, Instron Co., Norwood, MA) with a 50-kg load range at a crossed speed of 50 mm/min with tibia supported on a 3.35 cm span. Tibias were dried at 102°C in a drying oven for 24 h and weighed for dry matter determination. The dried tibia samples were extracted with a Soxhlet automatic apparatus (Gerhardt, Bonn, Germany) for 24 h to measure the fat-free tibia. The fat-free tibia samples were then ashed at 600°C for 3 h and re-weighed.

### Measurements of Nutrients Digestibility and N Balance

During the last 7 d of each phase (18 wk for phase 1 and 32 wk for phase 2), the total fecal collection method was used to measure the apparent total tract digestibility (**ATTD**) of nutrients and N balance. Seven replicates per treatment and 2 birds per replicate were employed. Birds were allowed ad libitum access to feed and water. Feed intake was measured, and fecal dropping was quantitatively collected twice a day for 3 consecutive days. Excreta samples collected were pooled per replicate and stored at −20°C until drying. Excreta samples were dried in a forced-air drying oven at 65°C for 72 h and finely ground for chemical analyses. Feed and excreta samples were analyzed for dry matter (method 930.15; AOAC, 2005), CP (method 990.03; AOAC, 2007), ether extract (method 920.39; AOAC, 2007), and ash (method 942.05; AOAC, 2007). Nitrogen balance including intake, excretion, and retention as total, egg, or body was calculated based on a study proposed by Barzegar et al. (2019).

### Measurements of Odor Emission and Volatile Fatty Acid in Fecal Samples

During the last week of each phase (18 wk for phase 1 and 32 wk for phase 2), odor emission was measured using the modified flux chamber method (Eklund, 1992). In brief, freshly voided excreta samples weighing approximately 150 g were placed in a 6 L plastic box. To make a concentration equilibrium of odor substances, 1 L/min of N gas (99.99%) was injected into the plastic box for 10 min. The emitted odor was measured using a Gastec detector (GV-100S, Gastec Co., Kanagawa, Japan) equipped with gas detector tubes for carbon dioxide, ammonia, hydrogen sulfide, and trimethylamine.

The concentrations of volatile fatty acids (**VFA**) were measured with freshly voided fecal droppings. At the end of the experiment, 1 g of fresh excreta samples was added to 9 mL of distilled water and mixed using a vortex mixer. The mixture was added with 0.05 mL of saturated HgCl_2_, 1 mL of 25% H_3_PO_4_, and 0.2 mL of 2% pivalic acid, and centrifuged (20 min, 1,000 × *g*, and 4°C). Then, the 1 mL of supernatant was used to measure the concentrations of VFA in excreta samples by the gas chromatography (6890 Series GC System, Hewlett Packard, Palo Alto, CA) as described by Lee et al. (2022).

### Measurements of Laying Performance and Egg Quality

In phase 2 (19–32 wk), egg weight and egg production (based on a hen-day basis) were recorded daily and used to calculate the egg mass, and ages at first egg production and to reach 50% egg production. The percentage of soft and broken eggs was calculated as (total number of soft and broken eggs per replicate/total number of eggs per replicate) × 100. Broken eggs are referred to eggs with cracked shells and damaged membranes, resulting in the leakage of egg components from the eggs. Soft eggs are referred to eggs with no shell (shell-less) and eggs with very thin shells (soft-shelled). The feed conversion ratio (**FCR**; feed intake/egg mass) was calculated on a 4-wk basis.

On the last 3 consecutive days at 22, 26, and 32 wk, 6 intact eggs per replicate were collected for the egg quality measurement. Eggshell thickness (without shell membrane), eggshell strength, Haugh unit, and yolk color score were measured using a digital egg tester (DET6000, Nabel Co., Ltd., Kyoto, Japan). Yolk color was automatically graded on a scale of 1 to 16, with 1 being a very pale yellow and 16 being a dark orange. Eggshell color was measured by a shell color reflectometer (TSS QCR, Technical Services, and Supplies, York, UK) to provide a reflectance score from 0% (black) to 81.8% (white).

### Determinations of Corticosterone in Yolk Samples

Corticosterone was assayed to see whether different CP levels would affect stress responses in laying hens. The separated yolks were pooled (3 yolks/replicate) and homogenized, and 4 g of homogenized egg yolks were diluted with an equal volume of phosphate buffered saline in the falcon tube. Then 1 mL of the diluted yolk was mixed with an equal volume of ethanol, and the mixture was extracted at 37°C for 1 h in the incubator and then centrifuged (Kim et al., 2021). The supernatants were analyzed using a CORT ELISA kit (Enzo Life Science Inc., ADI-901-097, Farmingdale, NY) per the manufacturer's recommendation.

### Statistical Analysis

Data were analyzed using the GLM procedure of the SAS (SAS Inst. Inc., Cary, NC). The model included dietary treatment as a fixed variable. The least square means were calculated for each treatment. Preplanned polynomial contrasts were employed to determine linear and quadratic effects of CP levels in diets. An alpha level of 0.05 was used to determine statistical significance.

## RESULTS

In phase 1, final body weight, body weight gain, and daily feed intake were not altered by dietary CP levels (Table 2). Decreasing dietary CP levels linearly increased (*P* = 0.006) the concentration of triglyceride, but decreased (*P* = 0.036) that of high-density lipoprotein cholesterol in serum samples. Dietary CP levels failed to affect total cholesterol, glutamic oxaloacetic transaminase, glutamic pyruvic transaminase, total protein, uric acid, and albumen in pullets (Table 3). Decreasing CP levels in diets linearly and quadratically (*P* < 0.05) increased the relative weights of abdominal fat contents in pullets (Table 4). However, internal organs including liver, kidney, spleen, small intestine (duodenum, jejunum, and ileum), bursa of Fabricius, and reproductive organs (ovary, oviduct, and the number of large yellow follicles) were not affected by dietary CP levels. Villus height and crypt depth were quadratically increased with lowering the CP levels in diets (Table 5). Dietary CP levels tended to lower villus height to crypt depth ratios, but statistical significance was not reached (linear effect, *P* < 0.073). Tibia traits were altered by dietary CP levels in pullets (Table 6). In phase 1, decreasing dietary CP levels increased the ATTD of dry matter (linear effect, *P* < 0.05) and ether extracts (linear and quadratic effects, *P* < 0.05) (Table 7). The ATTD of crude ash tended to be increased (*P* = 0.081) as dietary CP levels decreased in phase 1. In phase 2, the ATTDs of dry matter, CP, and ether extract were linearly increased (*P* < 0.001) while that of crude ash linearly lowered (*P* < 0.001) as the CP level of diets decreased (Table 7). Lowering CP levels in the diets of pullets linearly lowered excreta output, N intake, and N excretion, but did not affect daily feed intake (Table 8). Similarly, excreta output, N intake, and N excretion were linearly decreased as the CP decreased in the diets of laying hens (Table 8). In phase 1, carbon dioxide concentrations were linearly lowered (*P* < 0.01) as the CP levels in diets decreased (Table 9). However, CP levels did not affect the concentrations of ammonia and trimethylamine in fecal droppings of pullets (Table 9). Hydrogen sulfide was not detected in most samples tested except for that in pullets fed a 14% CP diet. During phase 2, dietary CP levels did not affect the concentrations of carbon dioxide, ammonia, and trimethylamine in feces of laying hens. At this stage, trimethylamine was not detected in all groups (Table 9). The concentrations of VFA in fecal droppings were not altered by dietary CP levels in phases 1 and 2 although propionate concentration tended to be decreased (quadratic effect, *P* = 0.054) with increasing CP levels (Table 10). Growth performance (i.e., final body weight, body weight gain, and daily feed intake) was not affected by dietary CP levels in laying hens (Table 11). Egg weight linearly and quadratically decreased (*P* < 0.05) as dietary CP levels decreased (Table 11). However, dietary CP levels did not affect egg production, egg mass, and feed conversion ratio in laying hens. Ages at first egg production and to reach 50% egg production were not altered by dietary CP levels (Table 11). Dietary CP levels did not affect eggshell qualities including eggshell thickness, and eggshell strength in laying hens (Table 12). Eggshell color at 32 wk was quadratically lowered as dietary CP levels decreased. However, this effect was not noted at 22 and 26 wk. Haugh unit, an indicator of egg freshness, was linearly increased as CP levels decreased in the diets of laying hens, but this effect was only detected at 26 wk (*P* < 0.05). Dietary CP levels lowered yolk color at all ages (Table 12). The concentration of corticosterone in yolk samples at 22 wk was linearly decreased (*P* < 0.001) as dietary CP levels decreased. However, this CP-induced decrease in corticosterone by different CP levels was not noted in eggs laid at 26 and 32 wk.

**Table 2:** Effect of dietary protein levels on growth performance in pullets (phase 1; 13–18 wk).

Item	Protein levels, %				SEM	*P* values for contrasts	
	18	16	14	12		Linear	Quadratic
Initial body weight, g/hen	1,217	1,210	1,238	1,229	16	0.377	0.959
Final body weight, g/hen	1,599	1,616	1,624	1,633	22	0.279	0.859
Body weight gain, g/hen	382	406	386	404	18	0.561	0.859
ADFI, g/hen/d	68.49	67.33	69.32	67.25	0.60	0.515	0.450

Abbreviation: ADFI, average daily feed intake.

**Table 3:** Effect of dietary protein levels on serum parameters in pullets (phase 1; 13–18 wk).

Item	Protein levels, %				SEM	*P* values for contrast	
	18	16	14	12		Linear	Quadratic
Total cholesterol, mg/dL	112	106	110	117	4	0.333	0.128
Triglyceride, mg/dL	13.8	17.0	24.3	24.0	2.1	0.006	0.519
GOT, U/L	164	157	167	159	5	0.836	0.898
GPT, U/L	2.00	1.71	1.86	1.83	0.46	0.884	0.798
Albumin, g/dL	1.49	1.41	1.40	1.48	0.05	0.905	0.193
HDL cholesterol, mg/dL	83.6	77.8	72.7	63.8	5.1	0.036	0.797
Total protein, g/dL	4.10	4.06	3.97	4.03	0.13	0.650	0.726
Uric acid, mg/dL	3.37	3.20	2.75	2.67	0.35	0.131	0.905

Abbreviations: GOT, glutamic oxaloacetic transaminase; GPT, glutamic pyruvic transaminase; HDL, high-density lipoprotein.

**Table 4:** Effect of dietary protein levels on relative organ weight (g/100 g of BW) in pullets (phase 1; 13–18 wk).

Item	Protein levels, %				SEM	*P* values for contrast	
	18	16	14	12		Linear	Quadratic
Abdominal fat	2.18	2.20	2.36	3.15	0.13	<0.001	0.021
Liver	1.47	1.49	1.45	1.50	0.12	0.921	0.904
Kidney	0.60	0.59	0.55	0.59	0.02	0.617	0.353
Spleen	0.19	0.19	0.17	0.20	0.02	0.772	0.698
Duodenum	0.32	0.33	0.31	0.33	0.01	0.814	0.896
Jejunum	0.66	0.64	0.72	0.65	0.03	0.749	0.203
Ileum	0.51	0.48	0.49	0.46	0.02	0.080	0.791
Bursa of Fabricius	0.08	0.08	0.06	0.07	0.01	0.289	0.720
Ovary	0.08	0.08	0.08	0.09	0.08	0.637	0.823
Oviduct	0.08	0.07	0.07	0.07	0.15	0.233	0.332
Number of large yellow follicles	ND	1.14	0.42	1.29	0.58	0.235	0.807

Abbreviation: ND, not detected.

**Table 5:** Effects of dietary protein levels on ileal morphology in pullets (phase 1; 13–18 wk).

Item	Protein levels, %				SEM	*P* values for contrast	
	18	16	14	12		Linear	Quadratic
Villus height (VH), μm	1066	1301	1167	1040	33	0.212	<0.001
Crypt depth (CD), μm	153.4	181.3	186.1	169.5	7.4	0.158	0.014
VH to CD ratio	6.978	7.305	6.299	6.224	0.350	0.073	0.612

Abbreviations: CD, crypt depth; VH, villus height.

**Table 6:** Effects of dietary protein levels on tibia characteristics in pullets (phase 1; 13–18 wk).

Item	Protein levels, %				SEM	*P* values for contrast	
	18	16	14	12		Linear	Quadratic
Fresh weight, g/100 g body weight	0.61	0.60	0.63	0.61	0.35	0.740	0.526
Length, cm	8.83	8.92	9.08	8.83	0.83	0.708	0.080
Width, cm	0.91	0.88	0.92	0.89	0.88	0.758	0.990
Strength, kgf	23.0	22.7	22.8	23.3	1.6	0.891	0.814
Dry matter, %	67.4	66.9	65.3	67.8	2.3	0.961	0.546
Fat-free dry matter tibia weight, g	5.35	5.41	5.40	5.00	0.26	0.404	0.415
Ash, g	2.03	2.00	2.08	2.15	0.08	0.284	0.611
Ash/fat-free dry matter, %	37.9	37.3	38.8	36.7	3.1	0.892	0.822

**Table 7:** Effect of dietary protein levels on apparent total tract digestibility of nutrients in pullets (phase 1; 13–18 wk) and laying hens (phase 2; 19–32 wk).

	Protein levels (phase 1/2), %					*P* values for contrast	
Item	18/19	16/17	14/15	12/13	SEM	Linear	Quadratic
Phase 1, %							
Dry matter	70.7	73.1	76.1	75.6	1.1	0.002	0.199
Crude protein	49.2	53.7	58.0	57.0	4.6	0.191	0.553
Ether extracts	79.6	87.3	94.5	94.8	1.4	<0.001	0.032
Crude ash	13.3	13.6	17.9	16.2	1.1	0.081	0.990
Phase 2, %							
Dry matter	71.1	71.6	74.9	76.5	0.9	<0.001	0.526
Crude protein	48.8	44.7	57.2	58.8	2.0	<0.001	0.183
Ether extracts	89.7	93.5	95.3	96.1	1.0	<0.001	0.133
Crude ash	39.4	23.9	31.0	27.0	2.6	<0.001	0.186

**Table 8:** Effect of dietary protein levels on nitrogen (N) balance in pullets (phase 1; 13–18 wk) and laying hens (phase 2; 19–32 wk).

Item	Protein levels (phase 1/2), %				SEM	*P* values for contrast	
	18/19	16/17	14/15	12/13		Linear	Quadratic
Phase 1, g/hen/d							
Daily feed intake	97.4	96.8	98.8	87.9	6.2	0.346	0.413
Excreta	105	98.8	93.7	80.4	6.3	0.009	0.589
N intake	2.93	2.47	2.21	1.71	0.16	<0.001	0.899
N excretion	1.48	1.09	0.93	0.73	0.12	<0.001	0.447
N retention as total^1^	1.45	1.38	1.28	0.97	0.17	0.052	0.507
Phase 2, g/hen/d							
Daily feed intake	115	113	112	114	5	0.826	0.740
Excreta	115	117	100	95.9	3.6	<0.001	0.322
N intake	3.54	3.08	2.75	2.19	0.12	<0.001	0.703
N excretion	1.81	1.72	1.17	0.90	0.09	<0.001	0.334
N retention as total^1^	1.73	1.36	1.57	1.29	0.08	0.007	0.596
N retention as egg^2^	1.00	1.01	1.00	1.04	0.03	0.370	0.640
N retention as body^3^	0.73	0.40	0.57	0.25	0.07	<0.001	0.967

1 Difference between N intake and excretion.

2 Calculated based on the following equation: egg mass (g/hen/d) × N concentration in eggs (%), assumed N concentration in egg as 1.94% (Miranda et al., 2015).

3 Difference between N retention as total and egg.

**Table 9:** Effect of dietary protein levels on odor gas emission (expressed as ppm) in pullets (phase 1; 13–18 wk) and laying hens (phase 2; 19–32 wk).

Item	Protein levels (phase 1/2), %				SEM^1^	*P* values for contrast	
	18/19	16/17	14/15	12/13		Linear	Quadratic
Phase 1							
Carbon dioxide	970	930	700	620	95	0.008	0.836
Ammonia	16.3	15.3	21.0	20.3	2.1	0.142	0.967
Hydrogen sulfide	ND^2^	ND	0.14	ND	-	-	-
Trimethylamine	27.6	47.2	36.0	35.6	6.5	0.668	0.146
Phase 2							
Carbon dioxide	364	336	323	316	27	0.244	0.711
Ammonia	4.67	3.40	5.30	3.50	0.68	0.677	0.731
Hydrogen sulfide	ND	ND	ND	ND	-	-	-
Trimethylamine	9.50	7.75	8.75	5.75	2.80	0.430	0.827

Abbreviation: ND, not detected.

**Table 10:** Effect of dietary protein levels on volatile fatty acid concentrations (mM) in pullets (phase 1; 13–18 wk) and laying hens (phase 2; 19–32 wk).

Item, mM	Protein levels (phase 1/2), %				SEM	*P* values for contrast	
	18/19	16/17	14/15	12/13		Linear	Quadratic
Phase 1							
Acetate	25.5	25.4	24.5	25.6	1.1	0.931	0.580
Propionate	2.12	1.68	1.80	2.32	0.24	0.474	0.054
Butyrate	2.10	2.81	2.62	1.86	0.60	0.739	0.236
Total VFA	29.7	29.8	28.9	29.8	1.4	0.324	0.779
Phase 2							
Acetate	29.0	27.4	32.6	28.8	2.5	0.681	0.658
Propionate	1.80	1.55	1.79	1.11	0.40	0.305	0.580
Butyrate	1.48	0.72	1.36	1.24	0.20	0.930	0.138
Total VFA	32.3	29.7	35.8	31.0	2.7	0.852	0.678

Abbreviation: VFA, volatile fatty acid.

**Table 11:** Effect of dietary protein levels on growth and egg performances in laying hen (phase 2; 19–32 wk).

Item	Protein levels, %				SEM	*P* values for contrast	
	19	17	15	13		Linear	Quadratic
Growth performance							
Initial body weight, g/hen	1,599	1,616	1,624	1,633	22	0.279	0.859
Final body weight, g/hen	1,924	1,966	1,968	2,007	50	0.268	0.976
Body weight gain, g/hen	325	350	344	374	49	0.523	0.959
ADFI, g/hen/d	98.9	98.5	99.4	99.2	0.9	0.684	0.899
Egg performance							
Egg weight, g/egg	57.5	57.6	56.7	54.2	0.6	<0.001	0.030
Egg production, %	62.2	68.8	70.9	67.9	13.1	0.747	0.717
Egg mass, g/d	44.2	42.4	48.1	46.0	2.6	0.340	0.950
Feed conversion ratio, g/g	2.53	2.44	2.43	2.48	0.05	0.600	0.284
Soft and broken egg, %	0.65	0.94	1.55	1.30	0.31	0.133	0.426
Age of egg production							
First egg production, d	141	140	140	143	2	0.558	0.476
50% egg production, d	157	152	152	152	3	0.151	0.330

Abbreviation: ADFI, average daily feed intake.

**Table 12:** Effects of dietary protein levels on egg quality and yolk corticosterone concentrations in laying hen (phase 2; 19–32 wk).

Item	Protein levels, %				SEM	*P* values for contrast	
	19	17	15	13		Linear	Quadratic
Eggshell thickness, mm							
22 wk	0.375	0.374	0.386	0.395	0.009	0.247	0.306
26 wk	0.491	0.375	0.383	0.390	0.009	0.378	0.053
32 wk	0.366	0.380	0.384	0.372	0.008	0.501	0.122
Eggshell strength, kgf							
22 wk	5.35	5.23	4.81	5.14	0.21	0.291	0.303
26 wk	5.35	5.23	5.00	5.39	0.23	0.910	0.287
32 wk	4.58	4.62	4.49	4.61	0.15	0.982	0.808
Eggshell color¹							
22 wk	25.1	25.8	25.3	25.6	0.6	0.711	0.762
26 wk	23.2	22.9	24.2	24.0	0.5	0.100	0.977
32 wk	25.7	24.2	24.2	25.5	0.7	0.843	0.046
Haugh unit							
22 wk	87.2	90.8	87.4	93.6	2.3	0.141	0.566
26 wk	90.1	90.3	91.7	95.2	0.8	<0.001	0.057
32 wk	95.3	93.9	90.9	95.1	1.6	0.594	0.085
Yolk color							
22 wk	6.19	5.76	5.31	4.91	0.08	<0.001	0.872
26 wk	5.64	5.50	5.30	4.39	0.05	<0.001	<0.001
32 wk	5.74	5.63	4.90	4.41	0.06	<0.001	0.002
Yolk corticosterone, pg/g							
22 wk	47.5	44.0	23.2	23.4	4.6	0.001	0.728
26 wk	53.2	52.6	54.9	53.6	7.2	0.918	0.963
32 wk	47.6	36.4	35.3	37.6	4.9	0.295	0.270

¹ Eggshell color was measured using a shell color reflectometer to provide a reflectance value from 0% (black) to 81.8% (white).

## DISCUSSION

It is clear from this study that lowering dietary protein levels has no detrimental effects on body weight gain in pullets, which concurred with other studies (Hussein et al., 1996; Saleh et al., 2018; Oluwabiyi et al., 2022). This can be attributed that the constant concentrations of indispensable amino acids in all experimental diets would support growth although dietary CP levels in the pullet diets dramatically dropped from 18 to 12%.

Serum biological profiles and relative organ weights are widely used to reflect the nutritional and developmental status of laying hens (Poosuwan et al., 2010; Kim et al., 2022). In the present study, various serum parameters including total cholesterol, glutamic oxaloacetic transaminase, glutamic pyruvic transaminase, albumin, total protein, uric acid, and indices of internal, intestinal, and reproductive organs were not affected by decreasing CP levels in the diets of pullets, indicating that all birds on different CP diets performed well without nutritional deficiencies. In addition, uric acid in serum samples is often used as an indicator of amino acid deficiency or imbalance (Namroud et al., 2008). However, none of the protein-related serum parameters (i.e., total protein, albumin, and uric acid) were altered by different CP levels in diets. Our study is in line with earlier studies (Chauynarong et al., 2008; Xin et al., 2022) that low CP diets did not negatively affect serum biochemical and organ developments in pullets.

As expected, lowering CP levels in the diets of pullets linearly increased triglyceride concentration in serum samples and relative abdominal fat weights while linearly decreased high-density lipoprotein cholesterol concentration in serum samples. These results might be deduced from the difference in dietary energy to protein ratio. In this study, the experimental diets were isocaloric but differed in CP levels leading to increasing calorie to CP ratio with decreasing CP levels in diets. Although pullets consumed equal amounts of feeds (i.e., equal calorie consumption), they decreased protein consumption leading to decreasing protein per energy consumed. It has been reported that increasing energy to protein ratio increased triglycerides but decreased high-density lipoprotein cholesterol concentration in laying hens (Torki et al., 2014; Khaliq et al., 2016). Thus, this study confirms that low CP diets could activate lipid synthesis for fat deposition in pullets. Indeed, earlier studies found that low CP diets increased relative abdominal fat contents in pullets (Van Emous et al., 2013), laying hens (Rozenboim et al., 2016), and broiler chickens (Brandejs et al., 2022).

Intestinal development and morphology including villus height and crypt depth in pullets are critical for the efficient absorption of energy and nutrients (Mathivanan et al., 2006; Chiang et al., 2010). Tibia characteristics are an indicator of mineral metabolism, bone maintenance, and development in laying hens (Kim et al., 2020a). Dietary CP levels did not affect tibia characteristics, which agree with a previous study (Hassan et al., 2013). Therefore, it can be tentatively concluded that lowering dietary protein levels might not affect the development of tibia in growing pullets. As to gut morphology, it is known that low protein diets lowered villus height and increased gut permeability in chickens (Law et al., 2018; Barekatain et al., 2019) indicating deteriorating the histological traits of gastrointestinal tract. However, lack of effect of low protein diets on gut morphology was also reported (Incharoen et al., 2010). In this study, we found that lowering CP levels quadratically increased villus height and crypt depth in pullets, especially those fed on a 12% CP diet displaying shortest villus height and lowest villus height and crypt depth ratio. Of note, pullets received a diet containing 16% CP exhibited highest villus height and villus height to crypt depth ratio indicating the optimal gut histology. Thus, nutritional strategies that can improve gut histology (e.g., villus height) would be beneficial if low protein diets are widely implemented in poultry industry.

In this study, dietary CP levels affected nutrient utilization at all ages. Especially, lowering CP levels increased the ATTDs of dry matter and ether extract in phase 1 and those of dry matter, CP, and ether extract in phase 2. Although the digestibility of CP (*P* = 0.191) and crude ash (*P* = 0.081) tended to increase with decreasing CP levels in diets, statistical significance was not noted. On the other hand, the ATTD of crude ash linearly decreased with decreasing CP levels in the diets of laying hens. It is well known that crystalline AAs are highly digestible upon ingestion. In phases 1 and 2, crystalline AA was gradually added as CP levels decreased in the diets of pullets and laying hens leading to greater crystalline AA to feed-origin CP ratios. Therefore, it is postulated that increasing CP digestibility with decreasing CP levels in diets seen in this study is apparently dedicated to the greater crystalline AA to CP ratios. It is known that unsaturated vs. saturated fatty acids from fat ingredients are highly digestible (Krogdahl, 1985). Tallow contains dominantly saturated fatty acids and its fat digestibility ranges from 70 to 76% in chickens (Leeson and Summers, 2005). On the other hand, corn has more than 80% unsaturated fatty acids (Kim et al., 2020b) and its fat digestibility is reported to be between 88 and 95% in chickens (Ravindran et al., 2016). In phase 1, compared to high protein diets, low protein feed increased corn content by 7.79% but decreased tallow by 1.02%. Likewise, low protein feed increased corn content by 15.43% but decreased tallow by 1.23% in phase 2. It is thus assumed that improved fat digestibility with decreasing CP levels in the diets of pullets and laying hens could be related to the concomitant increase in polyunsaturated fatty acids. Indeed, Tancharoenrat et al. (2014) reported that fat digestibility was improved in chickens fed diets containing corn or soybean oils compared with beef tallow.

Dietary ash concentrations were decreased as the dietary CP concentrations decreased. Low CP-induced decrease in ash digestibility can be attributed in the greater contribution of basal endogenous losses of minerals in low ash diet compared with that in high ash diet (Adeola et al., 2016). It should be, however, pointed out that tibia characteristics including the fat-free ash contents and breaking strength were not altered in this study, indicating no negative effects by low CP levels on mineral metabolism.

Nitrogen in the excreta of laying hens is originated collectively from undigested dietary CP, basal endogenous losses, and microbial fermentation end-products (Soomro et al., 2018). It is reported that excreted N can cause eutrophication, nitrous oxide, and global warming (Aneja et al., 2006). Therefore, reducing N excretion is critical for sustainable poultry production. Low protein diet has been and will be a prominent strategy for reducing N excretion in excreta. In this study, N excretion was linearly decreased as the dietary CP levels decreased in pullets (phase 1) and laying hens (phase 2). Our findings are in line with previous studies (Novak et al., 2006; Soares et al., 2019; Alfonso-Avila et al., 2022). Decreasing N excretion with decreasing CP levels in diets can be attributed to either low N intake (Table 9). N retention (calculated as the difference between N intake and N excretion) decreased with decreasing CP levels in phases 1 and 2. Interestingly, N retention as eggs was not altered with different CP levels in diets in laying hens (phase 2). In any event, our study clearly shows that low CP diets can lower N excretion in a dose dependent manner.

Odor emission from excreta is a principal pollutant which is a critical environmental concern related to intensive farming systems (Wang et al., 2021). Odor is mainly produced via microbial fermentation on undigested nutrients, such as CP and carbohydrates. Uric acid and urea of excreta are the general N components present (Groot Koerkamp, 1994). In general, the N components present in manure are converted to NH_4_^+^ by bacterial urease, which can be volatilized into the environment as odor producing NH_3_ form (Choi and Moore, 2008). In addition, VFA is produced via anaerobic fermentation by gut microbiota on undigested carbohydrates and proteins in the large intestine and are often used as an indicator of the volatile organic compound (**VOC**) concentration in excreta (Zahn et al., 1997). Odor emission can be controlled by proper management and dietary intervention (Park and Kim, 2020). Nahm (2007) demonstrated that reducing CP levels in the diets lowered ammonia gas, VFA, and other odors in poultry manure. Park and Kim (2019) reported that hydrogen sulfide was decreased with low CP diets. However, in this study, lowering CP levels from 19 to 13% in diets did not affect VFA, ammonia, hydrogen sulfide, or trimethylamine emission in pullets and laying hens. In line with our study, Roberts et al. (2007) reported no significant effect by low CP diets on ammonia emission in laying hens. However, in phase 1, carbon dioxide production was linearly decreased with decreasing CP levels in diets. At this stage, this result is unclear which needs further research.

Dietary CP levels did not affect body weight, body weight gain, and average daily feed intake of laying hens in phase 2. Decreasing CP levels in diets decreased (linear and quadratic effects, *P* < 0.05) egg weight, but did not affect egg production, egg mass, and feed conversion ratio in laying hens. This finding corroborates with earlier studies reporting that lowering CP levels decreased egg weight (Keshavarz and Jackson, 1992; Summers and Leeson, 1994; Alagawany et al., 2020). In addition, it is well understood that nutritional factors including CP levels, supplemental methionine, energy levels, or fat contents are known to affect egg weight (Waldroup and Hellwig, 1995; Grobas et al., 1999; Safaa et al., 2008). However, low CP-mediated decrease in egg weight was not associated with the decline in egg production and egg mass indicating that low CP diets can be utilized in laying hens.

In this study, dietary CP levels did not affect egg quality including eggshell strength, and eggshell color although they tended to lower (quadratic effect, *P* = 0.053) eggshell thickness at 26 wk (but not at 22 and 32 wk) and eggshell color at 32 wk (but not at 22 and 26 wk). The quadratic effect by dietary CP levels on eggshell thickness and eggshell color observed in this study is not clearly understood and requires further investigation.

Haugh unit is considered an index of egg freshness and is affected by CP levels in diets as it relates to protein synthesis at the time of egg formation (Hammershøj and Qvist, 2001). In this study, decreasing dietary CP levels in the diets of laying hens linearly increased the Haugh unit at 26 wk while partially increasing it at 22 wk (*P* = 0.141). This finding indicates that protein synthesis for albumen during egg formation in the oviduct was not impaired due to dietary CP levels. On the other hand, Haugh unit at the early phase of laying hens was improved with decreasing CP levels in diets. This finding might be partly related to higher crystalline AA supplementation leading to higher systemic availability for albumen synthesis although dietary CP levels were lowered. Similarly, Shim et al. (2013) reported that lowering dietary CP levels increased Haugh unit.

Yolk color increased as the CP levels decreased in the diets in this study. This effect was secondary to the increase in corn gluten meal in low CP diets as explained elsewhere (Moros et al., 2002; Galobart et al., 2004). It is well known that corn gluten meal is rich in natural pigments (e.g., xanthophylls) for egg yolk coloration. However, it should be pointed out that decreasing CP levels inversely increased corn inclusion but lowered corn gluten meal. Our study suggests that corn gluten meal vs. corn is considered more effective feed ingredient to potentiate yolk pigmentation.

In this study, we attempted to investigate whether low CP diets would affect physiological stress responses in laying hens. We measured yolk corticosterone which is a noninvasive method for estimating the stress status of laying hens (Saino et al., 2005; Downing and Bryden, 2008). The concentration of yolk corticosterone was linearly decreased with decreasing CP levels in diets at 22 wk. However, this effect was not observed at 26 and 32 wk. It is generally understood that stress can cause the secretion of corticosterone in laying hens (Quinteiro-Filho et al., 2012). Indeed, corticosterone was elevated in laying hens raised under suboptimal conditions including high stocking density, heat stress, or immune suppression (Najafi et al., 2015; Nawab et al., 2020). Per the positive relationships between corticosterone concentration in eggs and blood samples (Engel et al., 2022), our finding might indicate that laying hens fed on low CP diets were in a less stressed state. On the contrary, Lee et al. (2016) reported that low CP diets (18 vs. 19% CP in diets) tended to increase serum concentration of corticosterone in broiler chickens. Thus, the low CP-induced decrease in yolk corticosterone warrants further studies.

It is concluded that low CP levels in diets did not affect growth performance, organ weights, and tibia characteristics although they impaired gut morphology in pullets. In addition, there was no significant effect by dietary CP levels on the days at first egg production and to reach 50% egg production in laying hens. Nitrogen excretion linearly lowered as dietary CP levels lowered in pullets and laying hens, which was associated with reduction in N intake. The digestibilities of dry matter and ether extract were linearly increased with decreasing dietary CP levels in pullets and laying hens. Low CP diets decreased egg weight but increased Haugh unit in laying hens. Finally, yolk corticosterone at 22 wk, but not at 26 and 32 wk, were decreased with decreasing dietary CP levels in laying hens. Collectively, our study shows that dietary CP could be lowered without adversely affecting the performance of pullets and laying hens. Furthermore, lowering CP levels in diets is considered beneficial as manifested by increased nutrient digestibility, higher Haugh unit, and lowered yolk corticosterone in laying hens. Future studies are warranted to see whether dietary intervention (e.g., feed additives or functional amino acids) would help prevent the low CP-mediated reduction in intestinal morphology.
